# Insights Into the Role of CircRNAs: Biogenesis, Characterization, Functional, and Clinical Impact in Human Malignancies

**DOI:** 10.3389/fcell.2021.617281

**Published:** 2021-02-05

**Authors:** Sabah Nisar, Ajaz A. Bhat, Mayank Singh, Thasni Karedath, Arshi Rizwan, Sheema Hashem, Puneet Bagga, Ravinder Reddy, Farrukh Jamal, Shahab Uddin, Gyan Chand, Davide Bedognetti, Wael El-Rifai, Michael P. Frenneaux, Muzafar A. Macha, Ikhlak Ahmed, Mohammad Haris

**Affiliations:** ^1^Functional and Molecular Imaging Laboratory, Cancer Research Department, Sidra Medicine, Doha, Qatar; ^2^Dr. B. R. Ambedkar Institute Rotary Cancer Hospital (BRAIRCH), All India Institute of Medical Sciences (AIIMS), New Delhi, India; ^3^Research Branch, Sidra Medicine, Doha, Qatar; ^4^Department of Nephrology, All India Institute of Medical Sciences (AIIMS), New Delhi, India; ^5^Diagnostic Imaging, St. Jude Children’s Research Hospital, Memphis, TN, United States; ^6^Department of Radiology, Perelman School of Medicine at the University of Pennsylvania, Philadelphia, PA, United States; ^7^Dr. Rammanohar Lohia Avadh University, Ayodhya, India; ^8^Translational Research Institute, Academic Health System, Hamad Medical Corporation, Doha, Qatar; ^9^Department of Endocrine Surgery, Sanjay Gandhi Post Graduate Institute of Medical Sciences, Lucknow, India; ^10^Laboratory of Cancer Immunogenomics, Cancer Research Department, Sidra Medicine, Doha, Qatar; ^11^Department of Internal Medicine and Medical Specialties, University of Genoa, Genoa, Italy; ^12^College of Health and Life Sciences, Hamad Bin Khalifa University, Doha, Qatar; ^13^Department of Surgery, University of Miami Miller School of Medicine, Miami, FL, United States; ^14^Academic Health System, Hamad Medical Corporation, Doha, Qatar; ^15^Watson–Crick Centre for Molecular Medicine, Islamic University of Science and Technology (IUST), Pulwama, India; ^16^Center for Interdisciplinary Research and Innovations, University of Kashmir, Srinagar, India; ^17^Laboratory Animal Research Center, Qatar University, Doha, Qatar

**Keywords:** circRNA, RNA binding protein, miRNA sponges, signaling pathways, tumor, drug resistance

## Abstract

Circular RNAs (circRNAs) are an evolutionarily conserved novel class of non-coding endogenous RNAs (ncRNAs) found in the eukaryotic transcriptome, originally believed to be aberrant RNA splicing by-products with decreased functionality. However, recent advances in high-throughput genomic technology have allowed circRNAs to be characterized in detail and revealed their role in controlling various biological and molecular processes, the most essential being gene regulation. Because of the structural stability, high expression, availability of microRNA (miRNA) binding sites and tissue-specific expression, circRNAs have become hot topic of research in RNA biology. Compared to the linear RNA, circRNAs are produced differentially by backsplicing exons or lariat introns from a pre-messenger RNA (mRNA) forming a covalently closed loop structure missing 3′ poly-(A) tail or 5′ cap, rendering them immune to exonuclease-mediated degradation. Emerging research has identified multifaceted roles of circRNAs as miRNA and RNA binding protein (RBP) sponges and transcription, translation, and splicing event regulators. CircRNAs have been involved in many human illnesses, including cancer and neurodegenerative disorders such as Alzheimer’s and Parkinson’s disease, due to their aberrant expression in different pathological conditions. The functional versatility exhibited by circRNAs enables them to serve as potential diagnostic or predictive biomarkers for various diseases. This review discusses the properties, characterization, profiling, and the diverse molecular mechanisms of circRNAs and their use as potential therapeutic targets in different human malignancies.

## Introduction

Circular RNAs (circRNAs) are single-stranded non-coding RNAs that are covalently linked to form a continuous closed-loop and participate in the regulation of transcriptional and post-transcriptional gene expression (Wang M. et al., 2017). In recent years, circRNA has become a hotspot in research due to its ability to regulate a myriad of processes that include transcription, translation, splicing and sequestering RNA binding proteins (RBPs) and microRNAs (miRNAs) from their targets (Bartsch et al., 2018). Apart from the widely accepted role of circRNAs as miRNA sponges, circRNAs are also found to act as protein sponges, scaffolds, decoys and recruiters (Huang A. et al., 2020). Studies have found that circRNAs promote tumor progression in cancers such as lung adenocarcinoma, gastric cancer and cervical cancer by acting as RNA sponge and binding to miRNA, thereby increasing downstream gene expression (Tang Q. et al., 2019; Zhang X. et al., 2019; Xu Y. et al., 2020). CircRNAs are formed in the circular transcript by backsplicing of premature messenger RNAs (mRNAs). During the transcription process in eukaryotic cells, there is always a competition between linear and backsplicing. The presence of long introns, RBPs and inverted repeat elements favor the backsplicing event during transcription, and the splice-donor site downstream is brought closer to the splice-acceptor site upstream either by RBP dimerization or by base pairing between inverted repeat elements (Kristensen et al., 2019). The backsplicing event can result in the formation of different types of circRNA such as exon-intron circRNA (ElcircRNAs) (consists of both introns and exons), circular intronic RNAs (formed by introns), exonic circRNA (formed by the splicing of introns), and tRNA intronic circRNA (formed by pre-tRNA splicing) (Zhao X. et al., 2019). CircRNAs are presumably more stable than linear RNA because of the lack of 5′ and 3′ends, and ribonucleases do not easily digest them. The short half-life of linear RNA can be overcome by constructing engineered circRNAs and cyclizing mRNA, thereby promoting stable protein expression in eukaryotic cells (Wesselhoeft et al., 2018). The expression of circRNAs is disrupted in a wide range of diseases, including cancer. They have been proposed as potential biomarkers for cancer therapy as circRNAs can be easily detected in the patients’ blood plasma (Wu Q. et al., 2019). circRNAs regulate cancer progression and are involved in various cancer signaling pathways such as PI3K/AKT, MAPL/ERK1/2, and Wnt/β-catenin signaling pathways due to their interaction with miRNAs (Yang Z. et al., 2017). The aberrant translation of circRNAs alters tumor malignancy, and in addition to the many described functions of circRNA, they can also be retro-transcribed and function as competitive RNA (Dong et al., 2016).

Circular RNAs were initially thought to be unable to translate through cap-dependent mechanisms due to their lack of 5′ cap structure and poly-A tail. But recent studies have shown the ability of circRNAs to translate in prokaryotes by mimicking DNA rolling circle amplification and association of circRNAs with translating ribosomes and the ability of circRNAs to generate proteins from circRNA minigenes (Abe et al., 2013; Pamudurti et al., 2017). The translations of circRNAs can be classified as an internal ribosome entry site (IRES) independent dependent and IRES dependent (Tatomer and Wilusz, 2017). IRES-independent translations are found in the circRNAs present in the HeLa cells (Abe et al., 2015). While IRES-dependent translations require additional non-canonical cellular factors to recruit ribosomes to the IRES element and are found in circZNF609 as the UTR element of circZNF609 drives the IRES-dependent translation process through splicing event (Legnini et al., 2017). Cap-dependent translation is inefficient and inhibited under stress conditions or viral infections. In contrast, mRNA translation can be initiated by an IRES-mediated cap-independent mechanism, which is known to be unaltered by these unfavorable conditions (Yang and Wang, 2019).

## Biogenesis of CircRNAs

**FIGURE 1:**
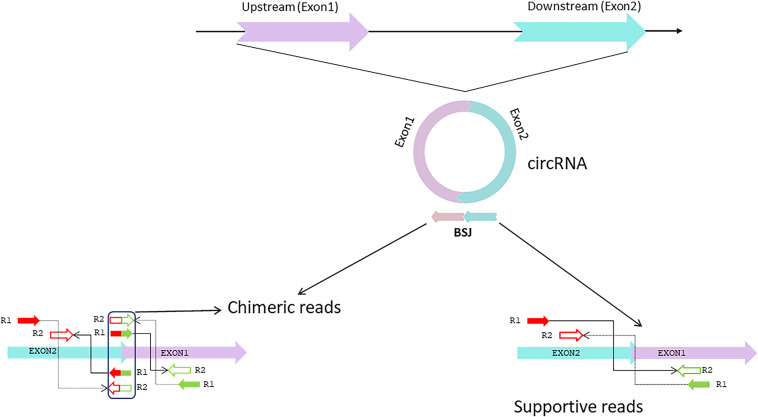
Biogenesis of circular RNA. The figure represents three different circular RNAs; Intron –exon, exon and intron circular RNAs, and its biogenesis mechanism during pre-mRNA splicing events. Intron pairing takes place with repeated inverted elements like *Alu* repeats, whereas lariat-driven biogenesis produces circRNAs during the exon skipping process. circRNAs can produce functional proteins that directly impact tumor progression (a few examples are shown in the box). circRNAs also act as protein sponges and are involved in protein recruitment.

Circular RNAs are produced by non- canonical splicing events commonly known as backsplicing, which is considered an alternative splicing event. Although back splicing is regarded as an alternative splicing event, the molecular mechanism underlying the circular RNAs’ biogenesis remains elusive (Mao et al., 2018). CircRNAs are derived from canonical splice sites and depend on canonical splicing machinery, which is usually inefficient to generate linear RNAs (Salzman et al., 2012; Jeck et al., 2013; Memczak et al., 2013; Ashwal-Fluss et al., 2014). On the contrary, results obtained from studies on *Drosophila* showed inhibition of spliceosome components by U2snRNP depletion or inhibition that caused increased circRNA generation compared to its linear counterparts (Liang D. et al., 2017). Hence it is proven that, when pre-mRNA processing events are halted, nascent RNA can be redirected to different alternative pathways that can facilitate back splicing and, ultimately, circRNA generation (Kramer et al., 2015; Liang D. et al., 2017). Apart from the defective splicing machinery, looping of flanking intron sequences on both sides of exons, namely splice donor site and splice acceptor site can support efficient circularization of diverse exons across eukaryotes (Kramer et al., 2015). The looping can be mediated by base pairing of *Alu* repeats or any inverted repeat elements located in the upstream and downstream introns (Ivanov et al., 2015; Kelly et al., 2015; Figure 1). Other mechanisms that facilitate backsplicing are the dimerization of RBPs like Quaking or FUS; those are known to bind in the specific motifs of the flanking introns (Conn et al., 2015; Errichelli et al., 2017; Verheijen and Pasterkamp, 2017). Even though most circRNAs are exonic, a large group of circRNA having long introns flanking the exons are involved in backsplicing (Jeck et al., 2013). These intron-exon circRNAs are usually derived from genes with highly active promoters (Enuka et al., 2016; Ferreira et al., 2018; Kristensen et al., 2018). Evidence suggests that most circular RNAs’ biogenesis was influenced by *cis-*acting elements and *trans-*acting splice factors (Kramer et al., 2015). Moreover, during the exon skipping process, lariat formation events can lead to the formation of intronic circRNA under specific circumstances like escaping from the debranching process of lariats (Kelly et al., 2015; Robic et al., 2020). Additionally, epigenetic modifications within the histones and gene bodies affect alternative splicing and directly impact circRNA biogenesis (Shukla et al., 2011; Bentley, 2014). These findings suggest that even though the biogenesis of circRNA is a product of inefficient canonical splicing, the introns within the circRNAs mostly spliced out during the process indicates biogenesis is instead a straightforward regulatory process orchestrated in the splicing machinery (Westholm et al., 2014).

## Properties and Characterization of CircRNAs

After the biogenesis of circRNAs, most of the exonic circRNAs transport to the cytoplasm, while the intronic and intron-exon circRNAs remain in the nucleus. The transportation of circRNAs is carried out in a size-dependent manner by ATP dependent RNA helicase URH49 (for shorter circles) and spliceosome RNA helicase UAP56 (for longer circles) (Huang et al., 2018). Exonic circRNAs generally have a low level of expression compared to their parental linear counterparts, except for a few whose expression levels are independent of their parental mRNA (Hsu and Coca-Prados, 1979; Capel et al., 1993; You et al., 2015; Holdt et al., 2016; Cortés-López et al., 2018; Vo et al., 2019). CircRNAs are exceptionally stable due to lack of free ends like mRNAs and are resistant to exonuclease digestion (Jeck et al., 2013; Memczak et al., 2013; Enuka et al., 2016). The turnover of circles is mostly mediated by N6-methyladonosine (m6A), and circles are subjected to the RNAse-P-multidrug associated protein (MRP) complex induced endonuclease cleavage (Liu C.X. et al., 2019). Another turnover mechanism is associated with miRNA regulated argonaute 2 (AGO2) protein-mediated cleavage, as shown in AGO2-miR7-miR-671 silencing complex for degradation of ciRS-7 (Hammond et al., 2001; Kleaveland et al., 2018). CircRNAs are enriched in exosomes or extracellular vesicles, suggesting that these extracellular vesicles might be a possible clearance mechanism or might serve as facilitators of the cell to cell communication through circles (Dou et al., 2016; Lasda and Parker, 2016; Preußer et al., 2018).

## Discovery and Profiling of CircRNAs

Circular RNAs are mostly evolutionary conserved and often expressed in cell-type, tissue-specific or developmental stages, specifically in organisms (Memczak et al., 2013). RNA sequencing in ribosomal RNA (rRNA) depleted total RNA leads to the discovery of many circRNAs in cancer cells, heart tissues and brain cells through a specific bioinformatics pipeline to identify back splicing junctions (Salzman et al., 2013; Maass et al., 2017). Individual circRNA identification and validations are explained in Table 1. Apart from the genome-wide proofing of circRNA using RNA sequencing or microarray analysis locus-specific circRNA profiling, validations can be done in a locus-specific manner using different methods, as explained in Table 2. The circRNAs can be visualized by RNA *in situ* hybridization and is a useful technique for understanding its biology by co-localizing potential miRNA sponges (You et al., 2015; Han et al., 2017).

**TABLE 1:** Selected published tools for circRNA detection and expression analysis.

Tool name	Software link	References
MapSplice	http://www.netlab.uky.edu/p/bioinfo/MapSplice2	Wang et al., 2010
find_circ	https://github.com/marvin-jens/find_circ	Memczak et al., 2013
segemehl	https://www.bioinf.uni-leipzig.de/Software/segemehl/	Hoffmann et al., 2014
CIRCexplorer	https://github.com/YangLab/CIRCexplorer	Zhang et al., 2014
circRNA_finder	https://github.com/orzechoj/circRNA_finder	Westholm et al., 2014
ACFS	https://github.com/arthuryxt/acfs	You et al., 2015
KNIFE	https://github.com/lindaszabo/KNIFE	Szabo et al., 2015
NCLscan	https://github.com/TreesLab/NCLscan	Chuang et al., 2016
PTESFinder	https://sourceforge.net/projects/ptesfinder-v1/	Izuogu et al., 2016
UROBORUS	https://github.com/WGLab/UROBORUS	Song et al., 2016
miARma	https://sourceforge.net/projects/miarma/	Andrés-León et al., 2016
circseq-cup	https://github.com/bioinplant/circseq-cup/	Ye et al., 2017
AutoCirc	https://github.com/chanzhou/AutoCirc	Zhou et al., 2017
CircPro	http://bis.zju.edu.cn/CircPro	Meng et al., 2017
PRAPI	http://forestry.fafu.edu.cn/tool/PRAPI/	Gao et al., 2018
circScan	https://github.com/sysu-software/circscan	Zhang and Yang, 2018
circTools	http://starbase.sysu.edu.cn/circTools.php	Zhang and Yang, 2018
STARChip	https://github.com/LosicLab/STARChip	Akers et al., 2018
CirComPara	http://github.com/egaffo/CirComPara	Gaffo et al., 2017
CircRNAFisher	https://github.com/duolinwang/CircRNAFisher	Jia et al., 2019
CircMarker	https://github.com/lxwgcool/CircMarker	Li et al., 2018g
circtools	https://github.com/dieterich-lab/circtools	Jakobi et al., 2019
NCLcomparator	https://github.com/TreesLab/NCLcomparator	Chen and Chuang, 2019
CIRI	https://sourceforge.net/projects/ciri/	Zheng Y. et al., 2019
CircRNAwrap	https://github.com/liaoscience/circRNAwrap	Li L. et al., 2019
Ularcirc	https://github.com/VCCRI/Ularcirc	Humphreys et al., 2019
Docker4Circ	https://github.com/kendomaniac/docker4seq	Ferrero et al., 2019

**TABLE 2:** Methods for detecting and quantifying circRNAs.

Detection method	Accuracy	Throughput	Advantage	Disadvantage	References
Northern blotting	Low	Low	Enzyme free detection at RNA level; high chance of identifying circular isoforms	Labor intensive	Salzman et al., 2012; Hansen et al., 2013; Rybak-Wolf et al., 2015
PCR+ sanger sequencing	High	Low	Experiment setup is easy; easy to identify isoforms	Only able to detect circular junctions	Ahmed et al., 2019; Karedath et al., 2019
RT-PCR	Medium to high	Medium	Analytically sensitive quantitative data	Less accurate in amplifying rolling circles	Ahmed et al., 2019; Karedath et al., 2019
Microarrays	Medium	High	Profiling of large number of circRNAs	Does not provide information about novel circles	Chen X. et al., 2018
RNA sequencing	Medium to high	Very high	High chance of identifying novel circles	Analysis requires computational power	Salzman et al., 2012; Okholm et al., 2017
ddPCR	High	Low	Digital quantification; eliminates the effect of rolling RT production circRNA quantification	Specialized machine required	Chen D.F. et al., 2018
Nanostring-nCounter	High	Medium to high	Enzyme free, digital quantification	Specialized machine required	Dahl et al., 2018

## Identification of CircRNAs From RNA-Seq Data Using Bioinformatics Approach

**FIGURE 2:**
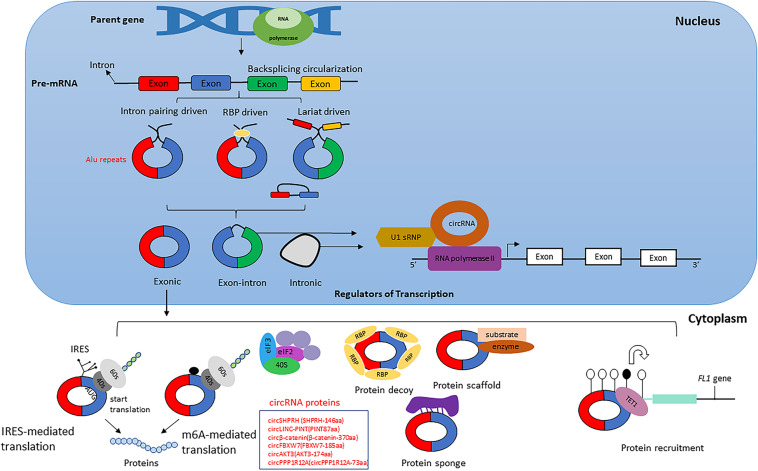
circRNA synthesis. The circRNA is formed by a head-to-tail backsplice junction (BSJ) through a downstream splice donor to an upstream splice acceptor. Chimeric reads align at the BSJ, directly confirming the existence of this non-canonical splice junction. The mates of the chimeric read can align to either of the BSJ forming exons. Supportive reads align to the BSJ forming exons in an orientation that is divergent regarding the direction of transcription but becomes properly inward-facing and convergent for BSJ. Adapted from Ahmed et al. (2016).

Several computational methods have been developed over the last few years to predict circRNA structures and their expression levels from rRNA-depleted or total RNA sequenced libraries (Table 1). RNA library preparation protocols can profile circRNA reads to varying extents. While methods such as polyA+ selection can result in a depleted representation of circRNAs (Jakobi and Dieterich, 2019), treatment with RNAse-R can enrich circRNA reads in the RNA-Seq data (Ahmed et al., 2016, 2019). The computational detection of circRNA expression requires a high sequencing yield from the high-throughput Next Generation Sequencing run as most circRNAs are expressed at low levels. Longer reads and paired-end libraries perform better for reliable detection of circRNA structure and estimation of expression. Pre-processing of RNA-Seq data such as removal of adaptor sequences or additional *in silico* flittering for rRNA reads can lead to performance enhancements of circRNA detection algorithms (Hansen, 2018; Jakobi and Dieterich, 2018). Indeed, all computational algorithms for circRNA detection use the unique morphology of circRNAs in which exons are atypically joined in a non-linear order through a head-to-tail “backsplice,” to identify chimeric reads that support the existence of the backsplice junction (BSJ). However, chimeric reads can also originate from multiple other sources such as genomic rearrangements of exonic sequences, tandem duplications, template switching from reverse transcription, chimeric amplification or *trans-*splicing (Ahmed et al., 2016; Jakobi and Dieterich, 2019). Although chimeric reads are the only reads that directly confirm the existence of a BSJ, the orientation of mapped paired-end reads across exon-junctions could be used in addition to inferring the presence of the BSJ. Thus, for each circRNA isoform, the expression can be summarized as the aggregate total number of chimeric and supportive reads that align to the exons in divergent orientation with respect to the direction of transcription, suggesting the presence of a BSJ instead of a linear junction (Figure 2).

Software tools for circRNA detection from RNA-Seq data use various approaches to identify BSJ and assess its expression (Table 1). While some methods use custom-scripts to analyze the data generated by the standard read mapping tools such as Bowtie2 (Langmead and Salzberg, 2012), TopHat2 (Kim et al., 2013), or STAR (Dobin et al., 2013), others have improved read alignment tools for circRNA detection with minor post-processing required (Hoffmann et al., 2014). Docker images have recently been developed that provide an end-to-end solution in a modular framework for multiple aspects of circRNA expression analysis such as circRNA prediction, classification, annotation, sequence analysis, and differential expression analysis (Gaffo et al., 2017; Ferrero et al., 2019). Also, multiple data resources have been built that provide annotation and functional information on circRNAs (Glažar et al., 2014; Chen et al., 2016; Xia et al., 2017; Li et al., 2018f; Meng et al., 2018) and their association with diseases and traits (Ghosal et al., 2013; Xia S. et al., 2018; Yao et al., 2018).

## Functional Role of CircRNAs With Special Emphasis on CircRNA-Protein Interaction in Tumor Cells

Circular RNAs have an important non-coding function as they retained the third position of codons, which is redundant and highly conserved in many circles (Memczak et al., 2013). The biological function of circRNAs is not yet fully identified except that a few circles act as miRNA sponges to regulate downstream target genes (Hansen et al., 2013; Memczak et al., 2013; Zheng et al., 2016; Piwecka et al., 2017). However, most circRNAs do not have multiple sponging sites that can effectively sponge miRNAs but contain protein binding sites (Ashwal-Fluss et al., 2014). For example, circRNAs viz. circMbl, circFOXO3, and circANRIL are involved in protein regulatory functions (Huang A. et al., 2020). Meanwhile, circPABPN1, located in the cytoplasm, acts as a protein decoy or a sponge to HuR, thereby suppressing PABPN1 gene translation and reducing cellular proliferation (Abdelmohsen et al., 2017). Some circRNAs act as protein scaffolds to regulate its parental gene function (Huang A. et al., 2020). CircFOXO3 acts as a protein scaffold that facilitates MDM2 independent ubiquitylation of p53 and acts as a sponge for MDM2, thereby preventing ubiquitination of its parental gene FOXO3 (Du et al., 2017a). These processes are highly implicated in cancer as the presence of circFOXO3 can induce stress-induced apoptosis and reduce cell viability (Du et al., 2017b). A study found that circAmotl1, a circRNA that is highly expressed in neonatal cardiac tissues, was involved in the cardioprotective role by binding to phosphoinositide-dependent kinase-1 (PDK1) and AKT-1 and facilitating the nuclear translocation of pAKT (Du et al., 2017a). Friend leukemia virus integration 1 (FLI1), an ETS transcription factor family member that acts as an oncogenic driver in hematological malignancies was found to enhance the invasiveness of breast cancer cells (MDA-MDB 231) by binding to the FLI1 promoter and recruiting TET1 (demethylase), thereby regulating DNA demethylation (Chen N. et al., 2018). Studies show that circRNAs like circZNF609, circFBXw7, circPiNT exon-2, and circSHPRH regulate tumor growth and act as a tumor promoter by producing functional proteins through template translation (Lin et al., 1986; Yang et al., 2018). CircFBXW7 contains an open reading frame with IRES that facilitates cap-independent translation (Yang et al., 2018). The protein product of circFBXW7 is termed as FBXW7-185aa, which is functionally active, and the overexpression of FBXW7-185aa protein (21-kDa) was found to inhibit tumor progression. In contrast, its knockdown induced a malignant phenotype *in vitro* and *in vivo* (Yang et al., 2018). Also, FBXW7-185aa reduced c-Myc half-life by altering its stabilization (Yang et al., 2018). In another study, the overexpression of protein SHPRH-146aa encoded by SNF2 histone linker PHD RING helicase (SHPRH) gene was found to reduce the malignant behavior and tumorigenicity of glioblastoma cells (U251 and U373) *in vivo* and *in vitro* (Zhang et al., 2018a). Another study showed an 87 amino acid peptide encoded by the long intergenic non-protein-coding RNA p53-induced transcript (LINC-PINT) suppressed glioblastoma cell proliferation *in vitro* and *in vivo* (Zhang et al., 2018b). This peptide directly interacts with the PAF1c complex and inhibits many oncogenes’ transcriptional elongation, thereby acting as a tumor suppressor (Zhang et al., 2018b). A recent study found that the production of a functional protein (termed as circPPP1R12A-73aa), which has a pro- tumorigenic function in colon cancer, is mediated by the activation of the Hippo-YAP signaling pathway (Zheng X. et al., 2019). Moreover, circβ-catenin and circAKT3 produce functional polypeptides β-catenin-370aa, and AKT3-174aa found to be implicated in tumorigenesis (Liang et al., 2019; Xia et al., 2019). Given the rapid evolution of circRNAs and their protein regulation role, we expect further studies in the next few years on circRNA translation and cancer-associated functional polypeptides.

## Role of CircRNAs in Tumorigenesis by Acting as miRNA Sponge

Many studies have highlighted the role of dysregulated circRNA in different diseases, including cancer (Liu et al., 2020). Dysregulation of circRNA occurs in various cancers, which provides a window of opportunity for its development as a viable therapeutic target. Different circRNAs have been reported to influence cellular signaling by binding with micro RNA (miRNA). It has been found that majority of circRNAs act as competitive endogenous RNAs (ceRNAs) and can modulate miRNA activity by binding to miRNA response elements (MREs) (Mitra et al., 2018). Furthermore, the binding of human antigen R (HuR) with many circRNAs in human cervical carcinoma HPV18 positive HeLa cell lines shows RBP binding sites in circRNAs (Abdelmohsen et al., 2017) and demonstrate the efficacy of circRNAs to scaffold the protein involved in the critical cellular phenomenon.

Similarly, another circRNA, circ-FOXO3, has been found to promote cellular senescence by interacting with ID-1, an anti-senescence protein and a stress pathway related proteins such as HIF1α and FAK, thus making these proteins unable to exert their functional roles (Du et al., 2017b). CircRNAs have been found to influence oncogenic phenomenon like promoting anchorage independent growth, cell proliferation, angiogenesis, metastasis, and drug resistance in different cancers (Su M. et al., 2019). Since circRNAs are dysregulated in different cancers, it is hypothesized that they can possibly be used as a molecular target as well as diagnostic and prognostic markers in both solid and hematological malignancies (Table 3). In this review, we will further discuss about different circRNAs specifically involved in oncogenesis.

**TABLE 3:** CircRNAs as diagnostic and prognostic markers in different cancers and their molecular targets.

Cancer type	Circular RNA	Source of sample	Expression in cancer	Molecular targets	Prognostic/Diagnostic potential	References
Breast cancer (BC)	circ-0005230	Tissues and cell lines	Upregulated	miR-618 and CBX8	Can serve as a prognostic predictor in BC patients	Xu Y. et al., 2018
	circKIF4A	Tissues and cell lines	Upregulated	miR-375	Can serve as a prognostic biomarker in TNBC^1^	Tang H. et al., 2019
	circGFRA1	Tissues and cell lines	Upregulated	miR-34a	Can serve as a diagnostic biomarker in TNBC	He et al., 2017
Bladder cancer	cTFRC	Tissues	Upregulated	miR-107	Can serve as a diagnostic biomarker in bladder cancer	Su H. et al., 2019
	circMTO1	Tissues	Downregulated	miR-221	Can serve as a prognostic biomarker in bladder cancer	Li et al., 2019b
Colorectal cancer (CRC)	circVAPA	Plasma	Upregulated	miR-101	Can serve as a diagnostic biomarker in CRC	Li X.N. et al., 2019
	circ RNA panel (circCCDC66, circABCC1, circSTIL)	Plasma	Downregulated	–	Can serve as a diagnostic biomarker in CRC	Lin et al., 2019
	hsa-circ-0004771	Circulating exosome	Upregulated	–	Can serve as a diagnostic biomarker in CRC	Pan et al., 2019
	circITGA7	Tissues and cell lines	Downregulated	miR-370-3p	Can serve as a diagnostic biomarker in CRC	Li et al., 2018h
	circCCDC66	Tissue	Upregulated	–	Can serve as a diagnostic biomarker in CRC	Hsiao et al., 2017
Gastric cancer (GC)	hsa-circ-0014717	Tissues and gastric juice	Downregulated	–	Can serve as a diagnostic biomarker in GC	Shao et al., 2017
	hsa-circ-00000520	Tissues, plasma and cell lines	Downregulated	Nine miRNAs and nine candidates mRNA that are predicted to have interaction with hsa_circ_0000520	Can serve as a diagnostic biomarker in GC	Sun et al., 2018
	hsa-circ-0000096	Tissues and cell lines	Downregulated	cyclin D1, CDK6, MMP-2 and MMP-9	Can serve as a diagnostic biomarker in GC	Lidonnici et al., 2017
	circNRIP1	Tissues and cell lines	Upregulated	miR-149-5p	Can serve both as a diagnostic and prognostic biomarker in GC	Zhang X. et al., 2019
	circPVT1	Tissues	Upregulated	miR-125	Can serve as a prognostic biomarker in GC	Chen J. et al., 2017
Hepatocellular carcinoma (HCC)	circSMARCA5	Tissues and plasma samples	Downregulated	–	Can serve as a prognostic biomarker in HCC	Li Y. et al., 2019
	circCDYL	Tissues	Upregulated	miR-892a and miR-328-3p	Can serve as a diagnostic biomarker in HCC	Wei et al., 2020
	circ104075	Tissues, cell lines and serum	Upregulated	miR-582-3p	Can serve as a diagnostic biomarker in HCC	Zhang et al., 2018d
	circ panel (hsa-circ-0000976, hsa-circ-0007750, hsa–circ-0139897)	Plasma	Upregulated	–	Can serve as a diagnostic biomarker in HCC	Yu et al., 2020
	circADAMTS13	Tissues	Downregulated	miR-484	can serve as a biomarker for evaluating the tumor burden of HCC patients	Qiu et al., 2019
	Cul2 circRNA (circ10720)	Tissues	Upregulated	Twist1, Vimentin	Can serve as a diagnostic biomarker in HCC	Meng et al., 2018
	SCD-circRNA2	Tissues	Upregulated	RBM3	Can serve as a prognostic biomarker in HCC	Dong et al., 2019
	circ101368	Tissues	Upregulated	miR-200a	Can serve as a prognostic biomarker in HCC	Li et al., 2018e
Lung cancer (LC)	circFARSA	Tissues and plasma	Upregulated	miR-330-5p and miR-326	Can serve as a diagnostic biomarker in NSCLC^2^	Hang et al., 2018
	hsa-circ-0075930	Cell lines and tissue	Upregulated	–	Can serve as a prognostic biomarker in NSCLC	Li et al., 2018d
	hsa-circ-0013958	Tissues, plasma and cell line	Upregulated	miR-134	Can serve as a diagnostic biomarker in LAC^3^	Zhu et al., 2017
	ciRS-7	Tissues	Upregulated	Promoted cell proliferation and growth	Can serve as a prognostic biomarker in NSCLC	Yan et al., 2018
	circPRMT5	Tissues and cell lines	Upregulated	miR-377, miR-382 and miR-498	Can serve as a diagnostic biomarker in NSCLC	Wang Y. et al., 2019
Osteosarcoma (OS)	circPVT1	Tissues, serum, and cell lines	Upregulated	ABCB1	Can serve as a diagnostic biomarker in OS	Kun-Peng et al., 2018b
	hsa-circ-0081001	Cell lines, tissues and serum	Upregulated	–	Can serve both as a diagnostic and prognostic biomarker in OS	Kun-Peng et al., 2018a
Ovarian cancer (OC)	circPLEKHM3	Tissues	Downregulated	miR-9, BRCA1, DNAJB6 and KLF4	Can serve both as a diagnostic and prognostic biomarker in OC	Zhang L. et al., 2019
	circWHSC1	Tissues	Upregulated	miR-145, miR-1182, MUC1 and hTERT	Can serve as a diagnostic biomarker in OC	Zong et al., 2019
Pancreatic cancer (PC)	circIARS	Tissues and plasma exosomes	Upregulated	miR-122	Can serve both as a diagnostic and prognostic biomarker in PC	Li et al., 2018b
	circPDE8A	Plasma exosomes	Upregulated	miR-338, MACC1	Can serve as a diagnostic biomarker in PC	Li et al., 2018i
	circLDLRAD3	Tissues, cell lines and plasma	Upregulated	–	Can serve as a diagnostic biomarker in PC	Yang F. et al., 2017
Esophageal cancer (EC)	circLPAR3	Tissues and cell lines	Upregulated	miR-198	Can serve as a diagnostic biomarker in EC	Shi et al., 2020
Gallbladder cancer (GBC)	circFOXP1	Cell lines and tissues	Upregulated	miR-370, PTBP1	Can serve as a prognostic biomarker in GBC	Huang et al., 2019

*^1^Triple-negative breast cancer. ^2^Non-small cell lung cancer. ^3^Lung adenocarcinoma.*

## Role of CircRNAs in Solid Malignancies

### Breast Cancer

Different circRNAs have been explored as diagnostic and prognostic markers in breast cancer (Wang and Fang, 2018). Using a microarray approach, Du et al. (2018) detected increased expression of a circRNA, circ-Dnmt1 in breast cancer *in vivo* and *in vitro*. Using different functional assays, they showed that circDNMT1 is involved in cell proliferation and survival by stimulating cellular autophagy (Du et al., 2018). At the molecular level, it was found that circDNMT1 interacts with both p53 and AU-rich element RNA-binding protein 1 (AUF1), thereby promoting their nuclear translocation (Du et al., 2018). In another study by Liang H.F. et al. (2017) circABCB10 was significantly upregulated in breast cancer tissues and was involved in the proliferation and inhibition of apoptosis in breast cancer cells. Furthermore, circABCB1O was found to sponge miR-1271 and promoted carcinogenesis through the circABCB10/miR-1271 axis (Liang H.F. et al., 2017).

Xu Y. et al. (2018) showed that circ-0005230 was upregulated in breast cancer tissues and found to be associated with adverse phenotypes in patients, thus could be used as a prognostic marker in breast cancer. At the molecular level, circ-0005230 was found to sponge miR-618, which regulated CBX8 expression, increasing cell migratory and invasive capabilities (Xu Y. et al., 2018). Tang H. et al. (2019) did the profiling of circRNAs in Triple-negative breast cancer (TNBC) using microarray and found circKIF4A to be the most upregulated circRNA. Additionally, circKIF4A was also associated with poor survival in TNBC patients (Tang H. et al., 2019). It was also found that circKIF4A promoted cell proliferation and migration by binding to miR-375 and regulated the expression of KIF4A *via* sponging miR-375 (Tang H. et al., 2019). Another study by He et al. (2017) found circGFRA1 to be significantly upregulated in TNBC cell lines and tissues. It was also shown that upregulated circGFRA1 correlated with poorer survival in TNBC patients. Moreover, the knockdown of circGFRA1 inhibited proliferation and promoted apoptosis in TNBCs (He et al., 2017). It was subsequently shown that circGFRA1 and GFRA1 regulated their expression by sponging miR-34a, thus highlighting the potential of circGFRA1 as a prognostic marker in TNBC (He et al., 2017).

### Bladder Cancer

Zhong et al. (2016) analyzed circRNA profile in bladder cancer using microarray and found that circTCF25 could sequester miR-103a-3p/miR-107, potentially leading to the upregulation of 13 targets related to cell proliferation, migration and invasion. It was demonstrated that the downregulation of miR-103a-3p and miR-107 could increase CDK6 expression and promote proliferation/migration of Bladder cancer cells *in vitro* and *in vivo*. This study suggests using circTCF25 as a potential therapeutic target in bladder cancer (Zhong et al., 2016). Zhong et al. (2017) performed a microarray profile of bladder carcinoma and found that circMYLK and VEGFA were significantly upregulated in bladder cancer. At the molecular level, it was found that circMYLK suppressed the activity of miR-29a and regulated the expression of VEGFA, thereby activating the VEGFA/VEGF R2 signaling pathway (Zhong et al., 2017). *In vitro* data indicated that circMYLK promoted epithelial-mesenchymal transition (EMT), cell proliferation and evasion of apoptosis by activating the Ras/ERK signaling pathway (Zhong et al., 2017). Another study by Su H. et al. (2019) found that the expression of circRNA cTFRC was upregulated and significantly correlated with poor prognosis of bladder cancer patients. Also, cTFRC was found to be a competing endogenous RNA (ceRNA) for miR-107 and mediated TGF-β-induced EMT in bladder cancer cells (Su H. et al., 2019).

Moreover, the downregulation of cTFRC inhibited the invasive potential of bladder cancer cells (Su H. et al., 2019). Indeed, this study highlighted the potential of cTFRC as a prognostic marker in bladder cancer (Su H. et al., 2019). In another study, Li Y. et al. (2019) explored the role of circRNA circMTO1 in bladder cancer. The expression levels of circMTO1 were downregulated in bladder cancer tissues, and reduced circMTO1 levels positively correlated with poor survival in bladder cancer patients. Besides, the overexpression of circMTO1 resulted in the inhibition of EMT, sponging of miR-221 and suppressing the E-cadherin/N-cadherin pathway (Li Y. et al., 2019). Overall, the study highlighted the potential of circMTO1 as a prognostic marker and a therapeutic target in bladder cancer (Zhong et al., 2017).

### Colorectal Cancer

Weng et al. (2017) analyzed the clinical significance of ciRS-7, a potential mIR-7 sponge in colorectal cancer (CRC). The study evaluated the effect of ciRS-7 on miR-7, and its target genes EGFR and RAF1. The levels of ciRS-7 were found to be upregulated in CRC tissues as compared to normal mucosae. Moreover, the increased expression of ciRS-7 in CRC cell lines (HCT116 and HT29) led to the blocking of miR-7 tumor-suppressive effects and an aggressive oncogenic phenotype (Weng et al., 2017). This study highlighted the efficacy of ciRS-7 as a promising prognostic biomarker and a potential therapeutic target in CRC patients (Weng et al., 2017). Another study by Zeng et al. (2018) found that circHIPK3 was significantly upregulated in CRC tissues and cell lines and positively correlated with metastasis and advanced clinical stage in CRC patients. Molecular analysis indicated that attenuation of circHIPK3 inhibited cell proliferation, migration, invasion, and induced cell death in CRC cell lines (Zeng et al., 2018).

Moreover, circHIPK3 was found to sponge tumor suppressor miR-7, and the overexpression of circHIPK3 effectively reversed the inhibition of CRC cell proliferation induced by miR-7, thereby demonstrating the tumor-promoting role of circHIPK3 in CRC (Zeng et al., 2018). Furthermore, the silencing of circHIPK3, combined with miR-7 overexpression resulted in cell proliferation inhibition in CRC xenograft animal models (Zeng et al., 2018). Overall, the study indicated the tremendous potential of circHIPK3 as a prognostic marker and therapeutic target in CRC (Zeng et al., 2018). A recent study by Zhang et al. (2018c) found hsa_circ_0007534 to be significantly upregulated in CRC tumor tissues and correlated with tumor stage and lymph node metastasis. Moreover, the silencing of hsa_circ_0007534 resulted in apoptosis and reduced proliferation in CRC cell lines (Zhang et al., 2018c). Another study showed the upregulation of circRNA hsa-circ-0020397 and the downregulation of miR-138 in CRC cells (Zhang X.L. et al., 2017). Furthermore, hsa-circ-0020397 was found to antagonize the activity of miR-138 by influencing its target genes like telomerase reverse transcriptase (TERT) and programmed death-ligand 1 (PD-L1) leading to increased cell proliferation and invasion in CRC (Zhang X.L. et al., 2017).

Many circRNAs in CRC have been shown to have diagnostic potential [reviewed in Hao et al. (2019)]. In a study by Li X.N. et al. (2019) circVAPA was found to be upregulated in the tissues and plasma of CRC and promoted CRC progression by sponging miR-101. CircVAPA promotes CRC cell proliferation, migration, invasion, and inhibit apoptosis in CRC cell lines (Li X.N. et al., 2019). Another study by Lin et al. (2019) evaluated the efficacy of using plasma circRNAs panel (circCCDC66, circABCC1 and circSTIL) as diagnostic markers in CRC. The plasma levels of the three circRNAs were found to be significantly reduced in CRC patients compared to healthy controls (Lin et al., 2019). Specifically, circCCDC66 and circSTIL were found to be efficient early-stage diagnostic markers in CRC (Lin et al., 2019).

Moreover, it was shown that combining the three circRNA panel with carcinoembryonic antigen (CEA) and carbohydrate antigen 19-9 (CA19-9) might improve CRC diagnosis (Lin et al., 2019). A study by Pan et al. (2019) found that exosomal circRNA hsa-circ-0004771 to be significantly upregulated in CRC patients’ sera. Moreover, the elevated expression of exosomal hsa-circ-0004771 in the serum of CRC patients was found to be tumor-derived and showed a high diagnostic value in CRC. Another study by Li et al. (2018h) found that circRNA circITGA7 and its host gene ITGA7 were downregulated in CRC tissues and cell lines. Also, circITGA7 was found to negatively regulate the proliferative Ras signaling pathway by binding to miR-370-3p and antagonizing its suppression of neurofibromin 1, a negative regulator of the Ras signaling pathway (Li et al., 2018h). Another study showed the upregulation and association of circCCDC66 with poor CRC prognosis (Hsiao et al., 2017). circCCDC66 was found to influence cell proliferation, migration, invasion, and anchorage-independent growth in CRC cells, demonstrating its potential as a CRC biomarker (Hsiao et al., 2017).

### Esophageal Squamous Cell Carcinoma

A study by Xia et al. (2016) found that hsa-circ-0067934 was overexpressed in Esophageal squamous cell carcinoma (ESCC) tissues. Additionally, high expression of hsa-circ-0067934 associated with poor differentiation, I-II T stage, and I-II TNM stage and promoted ESCC proliferation and migration. Moreover, the silencing of hsa-circ-0067934 by siRNA resulted in the inhibition of proliferation, migration, and blocking of the cell cycle in ESCC cells, thereby suggesting its potential as a biomarker and therapeutic target in ESCC (Xia et al., 2016). Another study found that circRNA, circLPAR3, to be highly expressed in ESCC tissues, upregulated *MET* gene expression by sponging miR-198 and promoted the migration, invasion and metastasis of ESCC cells through the activation of RAS/MAPK and PI3K/Akt signaling pathways, thus showing its potential as a diagnostic target in ESCC (Shi et al., 2020).

### Gastric Cancer

A study by Pan H. et al. (2018) highlighted the clinical significance of ciRS-7 in gastric cancer (GC) and found that the overexpression of ciRS-7 blocked miR-7-induced tumor suppression in GC cell lines and induced an aggressive phenotype by antagonizing PTEN/PI3K/AKT pathway. This study indicates the possibility of using cIRS-7 as a therapeutic target and a prognostic biomarker in GC (Pan H. et al., 2018). Another study reported circRNA-0023642 to be upregulated in GC tissues and cell lines (Zhou et al., 2018). The altered expression of circRNA-0023642 influenced the EMT pathway and its associated genes like N-cadherin, vimentin, snail, and E-cadherin. Moreover, the downregulation of circRNA-0023642 suppressed proliferation, migration, and invasion of GC cells, thereby suggesting circRNA-0023642 as a potential therapeutic target in GC (Zhou et al., 2018). Shao et al. (2017) did a global circRNA expression profile using microarray, and hsa-circ-0014717 was the most downregulated circRNA in GC. In addition, the expression levels of hsa-circ-0014717 were found to be associated with tumor stage, distal metastasis, tissue carcinoembryonic antigen (CEA) and carbohydrate antigen 19-9 (CA19-9) expression (Shao et al., 2017). Similarly, another circRNA hsa-circ-0000520 was found to be downregulated in GC tissues and plasma (Sun et al., 2018). It was further seen that the hsa-circ-0000520 level in GC tissues negatively associated with the TNM stage (Sun et al., 2018). Another study by Li et al. highlighted the diagnostic utility of circRNA hsa-circ-0000096, which was found to be downregulated in GC tissues and GC cell lines (Lidonnici et al., 2017). Moreover, the levels of cyclin D1, cyclin-dependent kinase 6 (CDK6), matrix metalloproteinases (MMPs) such as MMP-2 and MMP-9 were found to be significantly reduced both *in vitro* and *in vivo* in GC. Furthermore, the knockdown of hsa-circ-0000096 was shown to inhibit the proliferation and migration of GC cells *in vivo* and *in vitro* (Lidonnici et al., 2017). Another study by Zhang X. et al. (2019) found the expression of circNRIP1 to be significantly upregulated in GC. They found that knockdown of circNRIP1 blocked proliferation, migration, invasion, and the expression of AKT1 by sponging miR-149-5p and promoting oncogenesis in GC cells (Zhang X. et al., 2019). Thus, the inhibition of circNRIP1 can serve as a promising therapeutic target in GC (Zhang X. et al., 2019). Chen J. et al. (2017) identified a new circRNA circPVT1 that was found to be upregulated in GC tissues. CircPVT1 was found to sponge members of the miR-125 family and served as an independent prognostic marker for overall survival (OS) and disease-free survival (DFS) in GC (Li and Huang, 2017).

### Glioma

Li et al. (2018a) found hsa-circ-0046701 to be significantly upregulated in glioma tissue and cell lines. Moreover, hsa-circ-0046701 was found to sponge miR-142-3p and regulated the expression of Integrin alpha-V beta-8 (ITGB8) (Li et al., 2018a). A negative correlation was found between hsa-circ-0046701 and miR-142-3p expressions, indicating the role of hsa_circ_0046701/miR-142-3p/ITGB8 axis in the oncogenesis of glioma (Li et al., 2018a). Xu H. et al. (2018) studied the relationship between circNFIX and miR-34a-5p using CIRCexplorer2, circRNA-finder, CIRI, find-circ, and MapSplice2 in glioma. The study found circNFIX to be upregulated in glioma tissues and acted as a sponge for miR-34a-5p, thereby regulating its interaction with *NOTCH1* (Xu H. et al., 2018). Besides, an increased level of circNFIX promoted cell propagation and migration in glioma (Xu H. et al., 2018). An *in vitro* study found that silencing of circRNA, cZNF292 suppressed the proliferative and angiogenic potential of glioma cells. It was also found that cZNF292 resulted in cell cycle arrest at the S/G2/M phase with the downregulation of PRR11, Cyclin A, p-CDK2, VEGFR-1/2, p-VEGFR-1/2, and EGFR, proteins involved in cell cycle progression, thus implicating its role as a potential therapeutic target in glioma (Yang et al., 2016).

### Gall Bladder Cancer

Kai et al. (2018) studied the role of circHIPK3 in gall bladder cancer (GBC) and found elevated expression of circHIPK3 in GBC cell lines. The silencing of circHIPK3 inhibited the proliferation of gall bladder cell lines (Kai et al., 2018). Moreover, circHIPK3 sponged the tumor-suppressive miR-124 leading to an increased expression of miR-124 targets, including rho-associated protein kinase 1 (ROCK1) and CDK6, thereby showing the potential of circHIPK3 as a potential diagnostic marker and therapeutic target in GBC (Kai et al., 2018). Another study by Wang et al. found that the upregulation of circFOXP1 promoted cell proliferation, migration, and GBC invasion. Moreover, the study showed that circFOXP1 promoted the Warburg effect in GBC by interacting with polypyrimidine tract binding protein 1 (PTBP1) and promoting the expression of pyruvate kinase, liver, and RBC (PKLR). Additionally, the study also found that circFOXP1 promoted the Warburg effect in GBC by acting as a sponge of miR-370 that regulates the expression of PKLR, thus showing the potential of circFOXP1 as a prognostic biomarker in GBC (Wang S. et al., 2019). An interesting study by Huang et al. (2019) found that the increased expression of circERBB2 promoted GBC progression by regulating the nuclear localization of proliferation-associated protein 2G4 (PA2G4) and modulating ribosomal DNA transcription.

### Hepatocellular Carcinoma

Guan et al. (2018) studied circRNAs profile in hepatocellular carcinoma (HCC) using human circular RNA microarray and showed that hsa-circ-0016788 was found to be upregulated in both HCC tissues and cell lines. hsa-circ-0016788 was found to promote the proliferation and invasion of HCC through the hsa-circ-0016788/miR-486/CDK4 pathway (Guan et al., 2018). Another study by Meng et al. (2018) reported that Twist1 regulates vimentin expression by upregulating Cullin2 circRNA (circ10720), which absorbs miRNAs that target vimentin. Thus, the Culllin2 circRNA based mechanism involved in the Twist1-mediated regulation of vimentin during the process of EMT serves as a potential therapeutic target for the treatment of HCC (Meng et al., 2018). Another study found the expression of circSMARCA5 to be downregulated in HCC tissues and plasma samples (Li et al., 2019b). The overexpression of circSMARCA5 was found to inhibit proliferation, invasion, and increase apoptosis in HCC cells. Furthermore, circSMARCA5 correlated with tumor differentiation, TNM stage, cancer invasion, and cancer diameter in HCC (Li et al., 2019b). Wei et al. (2020) found circCDYL (chromodomain Y like) to be upregulated in HCC. CircCDYL was found to interact with mRNAs encoding hepatoma-derived growth factor (HDGF) and hypoxia-inducible factor asparagine hydroxylase (HIF1AN) by sponging miR-892a and miR-328-3p and influencing their downstream target (Wei et al., 2020). Furthermore, the authors demonstrated that circCDYL expression, combined with HDGF and HIF1AN, can function as independent markers for discrimination of early staged HCC (Wei et al., 2020). Thus, circCDYL provides a possibility for the early treatment of HCC (Wei et al., 2020). Zhang et al. (2018d) explored the diagnostic role of circ-104075, which was found to be highly upregulated in HCC tissues, cell lines, and serum. Besides, the expression of circ-104075 was found to be positively regulated by the hepatocyte nuclear factor 4 alpha (HNF4A), and circ-104075 acted as a ceRNA to upregulate YAP expression by sponging miR-582-3p (Zhang et al., 2018d). Yu et al. (2020) explored the diagnostic potential of hepatitis B virus (HBV) related HCC and plasma circRNAs. The study showed that plasma circRNA panel (circPanel) containing three circRNAs (hsa-circ-0000976, hsa-circ-0007750 and hsa-circ-0139897) could accurately detect Small-HCC, AFP-negative HCC, and AFP-negative Small-HCC (Yu et al., 2020). Qiu et al. (2019) found circADAMTS13, derived from Exon 13–14 of the *ADAMTS13* gene, to be downregulated in HCC tumor tissues. circADAMTS13 is negatively associated with tumor size but positively associated with prognosis in HCC (Qiu et al., 2019). circADAMTS13 interacted with miR-484 and served as a tumor suppressor during HCC progression by sponging miR-484 (Qiu et al., 2019). Dong et al. (2019) found that SCD-circRNA2 was significantly upregulated in HCC. SCD-circRNA2 was found to be regulated by RNA-binding protein 3 (RBM3), indicative of a short recurrence-free survival and poor overall survival in HCC patients (Dong et al., 2019). Another study by Li et al. (2018e) found that circ-101368 was upregulated in HCC tissue samples and the overexpression of circ-101368 correlated with poor prognosis in HCC. At the molecular level, knockdown of circ-101368 suppressed the migration and protein levels of high mobility group box 1 (HMGB1), receptor for advanced glycation end products (RAGE) and nuclear factor kappa-light-chain-enhancer of activated B (NF-κB) while increasing the E-cadherin expression in HCC (Li et al., 2018e).

### Lung Cancer

Zhang et al. (2018e) studied the expression of CDR1as, which is found to sponge miRNA-7 (miR-7). It was found that CDR1as levels increased with the development of non-small cell lung cancer (NSCLC) and negatively correlated with the expression of mIR-7 (Zhang et al., 2018e). Patients with high expression of CDR1 were found to exhibit high TNM stage, increased lymph node metastasis (LNM) and reduced overall survival (OS) (Zhang et al., 2018e). CDR1as was found to function as a miR-7 sponge to upregulate the target genes of miR-7 like EGFR, cyclin E1 (CCNE1), and phosphatidylinositol-4,5-bisphosphate 3-kinase catalytic subunit delta (PIK3CD) (Zhang et al., 2018e). Another circRNA, circ-0067934, was upregulated in NSCLC tissues and cell lines and its expression was significantly associated with TNM stage, lymph node status, and distant metastasis (Wang and Li, 2018). The study suggested that circ-0067934 is an EMT marker as it regulates EMT genes such as vimentin, N-cadherin, and E-cadherin (Wang and Li, 2018). Another study by Zhou et al. (2019) demonstrated the regulatory mechanism of circENO1 on its host gene enolase 1 (ENO1) and its role in glycolysis and tumor progression. The expression of both circENO1 and its host gene ENO1 was found to be upregulated in lung adenocarcinoma (LAC) cells. Mechanistically, the upregulated expression of ENO1 was due to the interaction of circENO1 as a ceRNA with miR-22-3p (Zhou et al., 2019). Moreover, the silencing of circENO1 resulted in the retardation of glycolysis and suppressed proliferation, invasion and EMT in LAC cells (Zhou et al., 2019). Dong et al. found that circFARSA, a circRNA derived from exon 5–7 of the FARSA gene, was found to be upregulated in NSCLC patients (Hang et al., 2018). *In vitro* experiments using NSCLC cell lines indicated that overexpression of circFARSA promoted cell migration and invasion (Hang et al., 2018). Moreover, *in silico* studies suggested that circFARSA might sponge miR-330-5p and miR-326 and relieve their inhibitory effects on oncogene fatty acid synthase (Hang et al., 2018). The expression of another circRNA, hsa-circ-0075930 was found to be upregulated in NSCLC cell lines and tissues (Li et al., 2018d). *In vitro* studies indicated that the depletion of hsa-circ-0075930 inhibited cell proliferation and induced cell cycle arrest and reversed EMT in NSCLC cells (Li et al., 2018d). Another study by Zhu et al. (2017) found hsa-circ-0013958 to be upregulated in LAC and associated with TNM stage and lymphatic metastasis. In addition, hsa-circ-0013958 was found to sponge miR-134, thereby upregulating the expression of cyclin D1, which has an oncogenic role in the development of lung cancer (Zhu et al., 2017). In a retrospective study, it was found that circRNA, ciRS-7 expression was elevated in NSCLC tissue and positively correlated with tumor size, lymph node metastasis, and TNM stage (Yan et al., 2018). Inhibition of ciRS-7 reduced proliferation and promoted cellular apoptosis in A549 lung cancer cell lines (Yan et al., 2018). Wang L. et al. (2018) studied the regulation and function of circRNA, hsa-circ-0008305 (circPTK2) in TGF-β-induced EMT and tumor metastasis in NSCLC. It was found that circPTK2 and TIF1γ were significantly downregulated in NSCLC cells undergoing EMT induced by TGF-β. CircPTK2 was found to function as a sponge of miR-429/miR-200b-3p, and miR-429/miR-200b-3p and influenced their downstream targets (Wang L. et al., 2018). Another circRNA, circPRMT5 was found to be highly expressed in NSCLC tissues and cell lines and positively correlated with large tumor size, advanced clinical stage, lymph node metastasis as well as poor prognosis (Wang Y. et al., 2019). At the molecular level, circPRMT5 simultaneously sponged three miRNAs (miR-377, miR-382, and miR-498) and alleviated their repression on the oncogenic enhancer of zeste homolog 2 (EZH2), thereby facilitating NSCLC progression (Wang Y. et al., 2019).

### Osteosarcoma

Many circular RNAs have been studied in osteosarcoma (OS) (Wan et al., 2020). circ-0016347 was found to act as a positive regulator of proliferation and invasion in OS cells, and it was identified as a sponge of miR-214 (Jin et al., 2017). The expression of another circRNA, circUBAP2, was found to be significantly increased in human OS tissues. circUBAP2 was found to promote osteosarcoma cell growth and inhibit apoptosis by inhibiting the expression of miR-143 and its downstream target B Cell Lymphoma 2 (Bcl-2) (Zhang H. et al., 2017). Another circRNA, circ-0009910, was overexpressed in OS and involved in the downregulation of miR-449a (Deng et al., 2018). Mechanistic studies have shown circ-0009910/miR-449a/IL6R axis to be a regulator of JAK1/STAT3 signaling pathway and a promoter of oncogenesis in osteosarcoma (Deng et al., 2018). Kun-Peng et al. (2018b) explored the role of circPVT1 in osteosarcoma and found that circPVT1 was significantly upregulated in OS. The knockdown of circPVT1 weakens the resistance to doxorubicin and cisplatin in OS cells by decreasing the expression of drug resistance gene ATP binding cassette subfamily B member 1 (ABCB1) (Kun-Peng et al., 2018b). Another circRNA, hsa-circ-0081001, was found to be significantly upregulated in the OS cell lines, tissues, serums and associated with poor overall survival and therefore demonstrated the potential of hsa-circ-0081001 as a potential prognostic or diagnostic biomarker for OS (Kun-Peng et al., 2018a).

### Ovarian Cancer

Chen Q. et al. (2018) found the expression of hsa-circ-0061140 to be upregulated in ovarian cancer (OC) cell lines. The knockdown of hsa-circ-0061140 was found to suppress cell viability and proliferation of OC. Mechanistically, hsa-circ-0061140 sponged miR-370 and suppressed the expression of FOXM1 by acting as a ceRNA of miR-370, thus promoting oncogenesis in OC (Chen Q. et al., 2018). Another circular RNA, circPLEKHM3 was found to be downregulated in OC tissues and peritoneal metastatic OC compared to primary ovarian carcinomas (Zhang X. et al., 2019). CircPLEKHM3 was found to function as a tumor suppressor in OC by sponging miR-9 to regulate the endogenous expression of breast cancer 1 (BRCA1), DNA heat shock protein family (Hsp40) member B6 (DNAJB6) and kruppel like factor 4 (KLF4) which consequently inactivates oncogenic AKT1 signaling (Zhang L. et al., 2019). Another study found the expression of circular RNA circWHSC1 to be upregulated in OC tissues (Zong et al., 2019). Mechanistically, circWHSC1 increased cell proliferation, migration and invasion, and inhibited cell apoptosis in OC (Zong et al., 2019). circWHSC1 sponges miR-145 and miR-1182 and upregulates the expression of downstream targets mucin 1 (MUC1) and human telomerase reverse transcriptase (hTERT) (Zong et al., 2019).

### Pancreatic Cancer

Li et al. (2018b) explored the role of circIARS in pancreatic ductal adenocarcinoma (PDAC) and found the expression of circIARS to be upregulated in the tissues and exosomes of the PDAC patients. CircIARS negatively correlated with miR-122 and ZO-1 and positively correlated with RhoA and RhoA-GTP levels, increased F-actin expression and focal adhesion, thereby promoting tumor invasion and metastasis, which suggests the role of circIARS as a prognostic marker in PDAC (Li et al., 2018b). Another circRNA, circPDE8A, was found to promote the invasive growth of PDAC cells by upregulating mesenchymal-epithelial transition (MET) and acted as a ceRNA for miR-338 to regulate metastasis-associated in colon cancer protein 1 (MACC1) and stimulate invasive growth of PDAC *via* the MACC/MET/ERK or AKT pathway (Li et al., 2018i). Furthermore, it was found that exosomal circPDE8A was associated with prognosis and disease progression in PDAC patients (Li et al., 2018i). Yang F. et al. (2017) found circLDLRAD3 to be upregulated in pancreatic cancer in addition to the significant association with venous invasion, lymphatic invasion, and metastasis.

## Role of CircRNAs in Haematological Malignancies

### Acute Myeloid Leukemia

Many circRNAs are associated with resistance in acute myeloid leukemia (AML) (Wu Z. et al., 2019). Shang et al. (2019) studied the role of circRNAs mediated chemoresistance in AML. Their study found that circPAN3 is upregulated in refractory and recurrent AML patient tissues and doxorubicin (ADM)-resistant THP-1 AML cell lines compared to non-transformed tissue and THP-1 AML cell lines (Shang et al., 2019). Mechanistically, circPAN3 decreased the expression of X-linked apoptosis protein inhibitor (XIAP) and further suggested that circPAN3 could be a crucial mediator of chemoresistance in AML cells by exploiting the circPAN3-miR-153-5p/miR-183-5p-XIAP axis (Shang et al., 2019). Another study by Yi et al. (2019) explored circvimentin (VIM) expression in AML patients and found it to be significantly upregulated in AML and correlated with WBC count. The study showed that circVIM expression could serve as a biomarker in differentiating AML patients from controls (Yi et al., 2019). Also, survival analyses in AML patients showed that over-expressed circVIM associated with shorter OS and leukemia-free survival (LFS) in whole-cohort AML, non-acute promyelocytic leukemia AML and cytogenetically normal-AML patients (Yi et al., 2019). Another study by Chen H. et al. (2018) found circANAPC7 to be most significantly upregulated in AML patients. The study predicted that circANAPC7 acts as a sponge for the miR-181 family and is associated with oncogenic pathways, thus demonstrating the potential of circANAPC7 as a potential biomarker for AML (Chen H. et al., 2018). Dong et al. explored the oncogenic potential of circular RNA DLEU2 (circDLEU2), microRNA 496 (miR-496), and Protein Kinase CAMP-activated catalytic subunit beta (PRKACB) in AML cell lines (Wu et al., 2018). CircDLEU2 was upregulated in AML tissues and promoted AML cell proliferation and inhibited cell apoptosis *in vivo* (Wu et al., 2018). Moreover, circDLEU2 was found to inhibit miR-496 expression and promote PRKACB expression. This study collectively indicated that circDLEU2 accelerated human AML by suppressing miR-496 and promoting PRKACB expression (Wu et al., 2018).

### Chronic Myeloid Leukemia

BCR-ABL1 is a fusion protein kinase derived from a reciprocal translocation between chromosomes nine and 22 and is a crucial protein for Chronic Myeloid Leukemia (CML) pathogenesis (Pan Y. et al., 2018). Tyrosine kinase inhibitors (TKIs) against BCR-ABL1 have revolutionized CML therapy; however, 25% of CML patients will switch TKIs at least once during their lifetime due to TKI intolerance or resistance (Bower et al., 2016), which is generally associated with mutations in the kinase domain (KD) of BCR-ABL1. Pan Y. et al. (2018) found that a novel circRNA named circBA9.3, derived from BCR-ABL1, can efficiently promote the proliferation and inhibit apoptosis of CML cells. Moreover, TKI resistance was associated with elevated circBA9.3 expression and positively correlated with the level of BCR-ABL1 (Pan Y. et al., 2018). Furthermore, the augmentation of cytoplasmic-ABL1 (c-ABL) and BCR-ABL1 oncoprotein expression by circBA9.3 suggests that circBA9.3 might serve as a therapeutic target in CML patients showing TKI resistance (Pan Y. et al., 2018). Another study by Liu et al. (2018) constructed an hsa-circ-0080145-mediated competing endogenous RNA (ceRNA) regulatory network. They found that hsa-circ-0080145 was found to regulate CML cell proliferation by sponging miR-29b.

## CircRNAs as Biomarkers

Numerous studies have demonstrated that circRNAs has emerging potential as a clinically relevant and disease-specific molecular biomarker in cancer and other complex diseases. Growing evidence suggests that circRNAs can be considered as promising biomarkers for the early diagnosis, metastasis, prognosis, and drug resistance of tumors due to their stable structure, the long half-life, tissue specificity, and abundance, and their presence in body fluids (Guo et al., 2014; Chen and Yang, 2015; You et al., 2015; Enuka et al., 2016; Yang Y. et al., 2017). CircRNAs are considered useful tools for the early detection of solid tumors, for example, hsa_circ_0043265 could be used as a biomarker for the early diagnosis of NSCLC due to its low expression in the early stages (Ren et al., 2020). The abundance of circMYLK can be useful as an early diagnosis tool and treatment of liver cancer (Li et al., 2019a).

**FIGURE 3:**
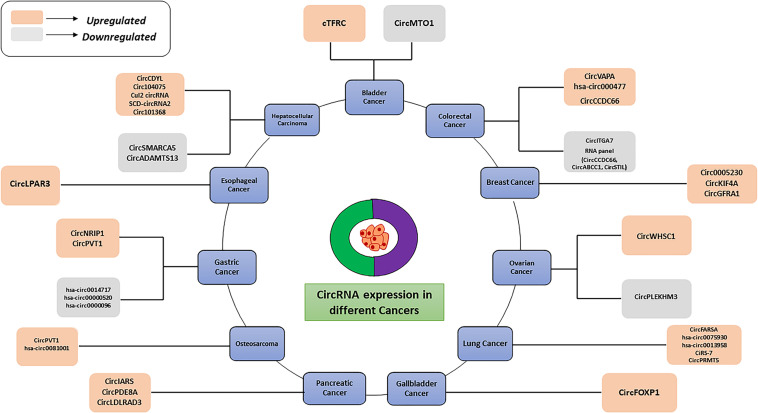
Graphical representation of circRNAs dysregulation in different cancers.

Moreover, circRNAs play a key role in tumor metastasis, Yang et al. (2020) found that the higher expression of circPTK2 can be positively correlated with a high survival rate in colorectal cancer patients. These findings suggest that circPTK2 may be a therapeutic target for metastatic colorectal cancer and a promising prognostic biomarker for early diagnosis. In PDAC, a high CircBFAR expression level was positively correlated with the tumor-node-metastasis (TNM) stage and was associated with poor prognosis. Hence, circBFAR could be used as a prognostic marker and therapeutic target for PDAC (Guo et al., 2020). Besides, many circular RNAs can be used as markers for drug resistance also. hsa_circ_0006528 (Gao et al., 2017), circMTO1 (Chen M. et al., 2019), circ_0001546 (Wu et al., 2020), and circ-LARP4 (Hu et al., 2020) exhibit abnormal expression levels in drug-resistant cells, suggesting that they could be used as diagnostic markers for drug resistance in tumors. CircRNAs have become accepted as biomarkers for multi-stage tumors in different cancer types. Thus far, many studies have demonstrated the potential of circRNAs as promising cancer biomarkers. The abnormal (upregulated or downregulated) expression levels of different circRNAs in various cancers are depicted in Figure 3. Recently studies have shown that circRNAs are also potential diagnostic markers for neurodegenerative diseases (Table 4). Several studies have indicated that circular RNAs are involved in repair and recovery after stroke, highlighting their potential therapeutic importance in regulating biological processes in brain injury due to ischemic stroke. Bai et al. (2018) demonstrated a critical role for circular RNA DLGAP4 (circDLGAP4) in repairing ischemic stroke brain damage. The gain of function of circDLGAP4 significantly reduced neurological deficits and decreased infarct areas and blood-brain barrier damage in a mouse stroke model. The study suggests that circDLGAP4 may serve as a novel therapeutic target for acute ischemic injury.

**TABLE 4:** CircRNAs as biomarkers in neurodegenerative diseases.

Disease type	Circular RNA	Expression level	References
Temporal lobe epilepsy	circ-EFCAB2	Upregulated	Li et al., 2018c
	circ-DROSHA	Downregulated	Li et al., 2018c
	circRNA-0067835	Downregulated	Gong et al., 2018
Multiple sclerosis	circ_0005402, circ_0035560	Downregulated	Iparraguirre et al., 2017
	hsa_circ_0106803	Downregulated	Cardamone et al., 2017
Alzheimer’s disease	CDR1as/ciRS-7	Downregulated	Akhter, 2018
	circPVT1	Downregulated	Panda et al., 2017
Parkinson’s disease	CDR1as/ciRS-7	Upregulated	Kumar et al., 2017
	circzip-2	Downregulated	Kumar et al., 2018
Ischemic stroke	circPDS5B	Upregulated	Zuo et al., 2020
	circCDC14A	Upregulated	Zuo et al., 2020
	circHECTD1	Upregulated	Han et al., 2018

Furthermore, a recently identified circRNA, *circHECTD1* can directly bind to *MIR142*, resulting in astrocyte activation and contributing to cerebral infarction. Silencing of *circHECTD1* expression can ameliorate cerebral infarction by inhibiting astrocyte activation (Han et al., 2018). These studies indicate that *circHECTD1* and circDLGAP4 could be envisioned as a novel biomarker and therapeutic target for stroke.

If circRNA detection methods can be effectively applied in clinical practice, they have the great potential to serve as early diagnostic markers to avoid patient distress in many life-threatening diseases.

## CircRNAs as Therapeutic Targets

High throughput deep RNA-sequencing followed by advanced bioinformatic analysis and functional characterization leads to discovering novel circRNAs with clinical significance. Researchers identified many circRNAs are related to cancer’s clinicopathological features, such as tumor metastasis, epithelial to mesenchymal transition, tumor stemness, and recurrence. Many studies have confirmed that circRNAs play a vital role in cancer initiation, progression, sensitivity to therapy, cancer stemness, and drug resistance. Many of the circRNAs identified can be used as a potential drug target in cancer, mainly due to their involvement in inducing therapy resistance *via* sponging miRNAs and alerting oncogenic signaling mechanisms. Numerous experiments have demonstrated that circRNAs play a regulatory role in drug resistance in many cancer types, and they can be used as potential drug targets. In lung cancer, CircPVT1 was found to mediate drug resistance *via* the miR-145-5p/ABCC1 axis. CircPVT1 knockout sensitizes tumor cells to conventional chemotherapeutic agents like cisplatin and pemetrexed (Zheng and Xu, 2020). This indicates that abrogating circRNAs in tumors can sensitize them to drugs effectively. Additionally, overexpression of circESRP1 and inhibition of the TGF-β signaling pathway can regulate the tumor response to chemotherapy in lung cancer (Huang W. et al., 2020). Circular RNAs viz., circ_0002483, and Circ_0076305 are involved in drug sensitivity in lung cancer *via* binding the target genes (Li X. et al., 2019). In breast cancer, regulating Hsa_circ_0006528, circKDM4C, and circMTO1 can effectively sensitize drug resistance in cancer types (Gao et al., 2017; Ma et al., 2019). In GC, CircPVT1 acts as a carcinogenic factor by mediating paclitaxel resistance by upregulating *via* ZEB1/miR-124-3p axis (Liu Y.Y. et al., 2019). CircFN1 regulates GC cell apoptosis through sponging miR-182-5p and promotes cisplatin resistance in GC, suggesting that circFN1 could be a potential therapeutic target (Huang X.X. et al., 2020). CircAKT3 and circCCDC66 are also involved in cisplatin resistance by altering target signaling pathways (Xu G. et al., 2020). In GC cells, apatinib resistance is mediated through circRACGAP1 *via* circRACGAP1/miR-3657/ATG7 axis (Ma et al., 2020). In colorectal cancer, circular RNA circ_0000338 has a tumor-suppressive effect and can enhance colorectal cancer cells’ chemosensitivity (Hon et al., 2019). Besides, a circular RNA, CIRS-122 (Wang et al., 2020) hsa_circ_0048234, was also involved in drug resistance in CRC (Xiong et al., 2017). Novel circular RNAs viz., hsa_circ_0000285, and circCdr1as, sensitize bladder cancer cells to cisplatin by restoring the expression of target genes (Chi et al., 2019; Yuan et al., 2019). In ovarian cancer, circCdr1as reduces cisplatin resistance by inhibiting miR-1270 (Zhao Z. et al., 2019). CircCELSR1 (hsa_circ_0063809) is upregulated in paclitaxel-resistant ovarian cancer tissues and cells (Zhang et al., 2020). Silencing of circCELSR1 may enhance the cytotoxic effect of paclitaxel in ovarian cancer cells suggesting circRNAs as promising therapeutic targets in many cancer types.

Surprisingly, many circRNAs exhibit an immunomodulatory function, as they are actively involved in immune regulation and autoimmune pathway regulation. Studies revealed that cells can recognize endogenous circRNA and *in vitro* synthesized circRNA by retinoic acid-inducible gene I (RIG-I), which can activate the autoimmune pathway (Chen Y.G. et al., 2017). It has been proved that cells can distinguish between endogenous circRNAs and exogenous or in vitro synthesized circRNAs by identifying the presence of N6-Methyladenosine modification (m6A) (Chen Y.G. et al., 2019). They further concluded that exogenous circRNAs without m6A modification could alter gene expression in the autoimmune pathway. Moreover, a recent study shows circRNAs can competitively bind double-stranded RNA-activated protein kinase (PKR) to extensively regulate cellular immune signaling pathways (Liu Y.Y. et al., 2019). Liu et al. (2016) identified differentially regulated circRNA, chondrocyte extracellular matrix-related circRNA (circRNA-CER) in osteoarthritis (OA), which can modulate the process of cartilage extracellular matrix injury *via* IL-1β and TNF-α regulation. This indicates that circRNA-CER may be a potential therapeutic target in osteoarthritis (OA) (Liu et al., 2016). Many studies indicate that circRNAs are implicated in Systemic lupus erythematosus (SLE), a chronic and incurable autoimmune disease. circRNAs are probably vital factors in SLE due to their functions as miRNA sponges. Wang X. et al. (2018) reported the downregulation of circIBTK in SLE, and they further revealed that circIBTK served as a miR-29b sponge to inhibit DNA demethylation and AKT signaling. Moreover, they have experimentally proved that the artificial overexpression of circRNAs can help reduce PKR activity in PBMCs in SLE patients, which is beneficial for treating SLE and other autoimmune diseases (Liu C.X. et al., 2019).

Many researchers confirmed that circRNAs play roles in bone marrow failure and hematologic malignancy. Xia P. et al. (2018) experimentally proved that circRNA cia-aGAS controls the balance between self-renewal and differentiation of hematopoietic stem cells (HSCs), and its deficiency disrupts the host homeostasis, leading to bone marrow failure and hematologic malignancy. This evidence suggests that circRNAs are involved in tumor stemness and can act as a possible therapeutic target.

Circular RNAs have also been shown to play vital roles in virus infections, providing a new strategy for developing vaccines against viruses, especially RNA viruses. The transfection of purified circRNAs generated *in vitro* into mammalian cells can induce the expression of innate immunity genes and thus protect against viral infection. This suggests that cells can distinguish endogenous and foreign circRNAs, dependent on the intron encoding the circRNAs (Chen Y.G. et al., 2017). In another study, Li et al. (2017) found that the antiviral function of circular RNA is universal and is mediated through NF90/NF110 release. Generally, the expression of circRNAs decreases during viral infection, so NF90/NF110 is released from circRNPs and binds to viral mRNAs to play an antiviral role.

Circular RNAs are extraordinarily enriched in the mammalian brain, and they are more abundant than their corresponding linear transcripts or parental mRNAs (Rybak-Wolf et al., 2015). Notably, circRNAs expressed in brain tissues have been reproducibly detected in human peripheral blood samples as they can pass through the blood-brain barrier (Rybak-Wolf et al., 2015). These findings suggest that the blood-borne circRNAs may be potential diagnostic biomarkers and reveal the pathophysiology for ischemic stroke and other brain-related diseases (Lu et al., 2020). Many circRNAs are upregulated in the mammalian brain during neuronal differentiation, indicating circRNAs are potentially involved in neurological disorders (Rybak-Wolf et al., 2015). CDR1as is an endogenous circRNA that is highly expressed in the human brain, acting as a miR-7 sponge, thus plays a key role in regulating Parkinson’s disease. Studies revealed that MiR-7 can modulate alpha-synuclein expression, a protein that always accumulates at the onset of Parkinson’s disease (Junn et al., 2009). This evidence strongly suggests that CDR1as may be a therapeutic target in Parkinson’s disease.

Additionally, CDR1as is implicated in Alzheimer’s disease by binding to miR-7, thereby reducing the ability of miR-7 to regulate ubiquitin-conjugating enzyme E2 A (UBE2A), a protein that decreases rapidly in Alzheimer’s disease (AD) and other neurological diseases (Akhter, 2018). Moreover, CDR1as inhibits the translation of NF-kβ, indirectly impairs protein functions that underlie AD’s development (Shi et al., 2017). As evidenced by the above studies and our conceptual understanding, circRNAs can be considered a potential biomarker and therapeutic target in AD diagnosis and treatment. Additionally, CDR1as is also implicated in cardiovascular diseases, mostly associated with myocardial infarction. Furthermore, CDR1as induces myocardial infarction by sponging mir7, protecting cardiac pericardium (Taïbi et al., 2014). Another novel circRNA, mitochondrial fission and apoptosis-related circRNA (MFACR), sponges miR-652-3p-MTP18 to mediate cardiomyocyte apoptosis, leads to mitochondrial fission, and ultimately promotes the development of myocardial infarction (Wang K. et al., 2017). Recent studies and current evidence indicate that circRNAs play a crucial role in controlling cellular dynamics in many types of tissue; thus, circRNAs may serve as a potential therapeutic avenue for many complex diseases, although further research is required for clinical applications to be feasible.

## Conclusion

Over the last few decades, the significance of circRNAs in numerous pathological processes has gradually been realized; while initially known as splicing redundant by-products, clinical studies have managed to uncover the hidden characteristics and ability of circRNAs to serve as ideal biomarkers in different pathologies. Unlike other RNA species, circRNAs have prognostic or diagnostic value due to high stability and specific cancer expression. Understanding the molecular mechanisms/regulatory roles of circRNAs in various transcriptional and translational processes in the eukaryotic machinery is essential to elucidate their human diseases’ roles, most importantly, cancer. Apart from the notorious role of circRNAs as miRNA sponges, further studies are required to reveal other mechanisms manifested by circRNAs. Although the expression of circRNAs has been extensively studied, their function remains inconclusive in normal physiological processes and human diseases. Many mechanistic aspects of how circRNAs potentially work in various cancers by affecting oncogenic pathways need to be better clarified, and we are still a long way from their development as a therapeutic or prognostic target that warrants thorough analysis of their mechanism of action. Research in this area must step away from simple quantification and therapeutic inference into mechanistic studies with a translational benefit. Developing technologies that enable sufficient single-cell level identification and modulation of circRNAs without disrupting their linear RNA partners may help gain more insight into the regulatory functions of circRNAs and thus help enhance the production of strategies that target the circRNA network in various human diseases. As circRNAs are generated from pre-mRNA back splicing, different bioinformatics algorithms have been designed to characterize circRNAs using different annotations to read back splicing sites. It will always make sense to develop the algorithm based on specific circRNA to avoid false reads and correlate the results using independent analysis by different platforms.

## Author Contributions

SN, AB, MS, IA, and TK: collected the literature, wrote the manuscript, and generated the figures. AR, SH, PB, RR, FJ, SU, GC, DB, WE-R, and MF: critical revision and editing of the scientific contents. AB, IA, TK, MM, and MH: conceived and designed the review contents and contributed to the manuscript writing and editing. All authors read and approved the final manuscript.

## Conflict of Interest

The authors declare that the research was conducted in the absence of any commercial or financial relationships that could be construed as a potential conflict of interest.
